# Rupture of the Uterus

**Published:** 1899-05-06

**Authors:** 


### RUPTURE OF THE UTERUS.

Mr. A. H. Davis[1] relates a case in which, on the termination of labour, rupture of the uterus was found to have occurred. Forceps had been applied but had slipped, podalic version had then been performed with great difficulty, the forceps being used for the after-coming head. The placenta, lying partly in the uterus and partly in the vagina, was then removed, and it was then that it was discovered that there was a rent anteriorly in the roof of the vagina and lower portion of the uterus. Two fingers passed through the rent, and a loop of gut could be felt between them. Laparotomy was performed two hours and a half after delivery; the abdomen contained a quantity of blood, the uterus was fairly contracted, the rent was about three inches long, beginning in the roof of the vagina and extending upwards and to the right through the wall of the uterus, the peritoneum being torn for some inches more than the uterine muscle, and gaping widely. The muscle was closed by interrupted catgut sutures, the peritoneum by continuous catgut. The abdomen was then irrigated with many pints of saline solution, all clots removed, the various cavities carefully sponged, and the incision in the parietes closed in layers. The patient made an excellent recovery.

[1] Brit. Med. Jour., April 22.

